# Prescription patterns and compliance with World Health Organization recommendations for the management of uncomplicated and severe malaria: A prospective, real-world study in sub-Saharan Africa

**DOI:** 10.1186/s12936-023-04650-y

**Published:** 2023-07-25

**Authors:** Vito Baraka, Abel Nhama, Pedro Aide, Quique Bassat, Agatha David, Samwel Gesase, Jonathan Gwasupika, Sebastian Hachizovu, Geofrey Makenga, Christian Ruchaho Ntizimira, Orikomaba Obunge, Kitoto Antoinette Tshefu, Marc Cousin, Nekoye Otsyula, Rashidkhan Pathan, Céline Risterucci, Guoqin Su, Christine Manyando

**Affiliations:** 1 National Institute for Medical Research (NIMR), Tanga Centre, Hospital Street, P.O Box 5004, Tanga, United Republic of Tanzania; 2 Instituto Nacional de Saúde (INS), Ministério da Saude, Maputo, Mozambique; 3 Centro de Investigação em Saúde de Manhiça, Manhica, Maputo Province Mozambique; 4 ISGlobal, Hospital Clínic—Universitat de Barcelona, Barcelona, Spain; 5 ICREA, Pg. Lluís Companys 23, 08010 Barcelona, Spain; 6 Pediatric Infectious Diseases Unit, Pediatrics Department, Hospital Sant Joan de Déu (University of Barcelona), Barcelona, Spain; 7 Consorcio de Investigación Biomédica en Red de Epidemiología y Salud Pública (CIBERESP), Madrid, Spain; 8 Nigerian Institute of Medical Research (NIMR), Lagos, Nigeria; 9 Tropical Diseases Research Centre, Ndola, Zambia; 10 Ipafu Rural Health Centre Chingola, Chingola, Zambia; 11 African Center for Research On End-of-Life Care, Kigali, Rwanda; 12 Center for Malaria Research and Phytomedicine (CMRAP), University of Port Harcourt, Port Harcourt, Nigeria; 13 The Hospital Center of Mont Amba Kinshasa, Kinshasa School of Public Health, University of Kinshasa, Kinshasa, Democratic Republic of Congo; 14 Novartis Pharma AG, Basel, Switzerland; 15 Novartis Pharma Services Inc., Nairobi, Kenya; 16 Novartis Healthcare Pvt. Ltd., Hyderabad, India; 17 Novartis Pharmaceuticals Corporation, East Hanover, NJ USA

**Keywords:** Compliance, Diagnosis, Malaria, Prescription, Recommendations, Treatment

## Abstract

**Background:**

This study aimed to evaluate the gap between guidelines and local clinical practice for diagnosis and treatment of uncomplicated and severe malaria, the patient characteristics, diagnostic approach, treatment, and compliance to standard guideline recommendations.

**Methods:**

This was a multicentre, observational study conducted between October 2020 and March 2021 in which patients of all ages with symptoms suggestive of malaria and who visited a healthcare facility were prospectively enrolled in six countries in sub-Saharan Africa (The Democratic Republic of the Congo, Mozambique, Nigeria, Rwanda, The United Republic of Tanzania, and Zambia).

**Results:**

Of 1001 enrolled patients, 735 (73.4%) patients had confirmed malaria (based on overall judgment by investigator) at baseline (uncomplicated malaria: 598 [81.4%] and severe malaria: 137 [18.6%]). Of the confirmed malaria patients, 533 (72.5%) were administered a malaria rapid diagnostic test. The median age of patients was 11 years (range: 2 weeks–91 years) with more patients coming from rural (44.9%) than urban (30.6%) or suburban areas (24.5%). At the community level, 57.8% of patients sought advice or received treatment for malaria and 56.9% of patients took one or more drugs for their illness before coming to the study site. In terms of early access to care, 44.1% of patients came to the study site for initial visit ≥ 48 h after symptom onset. In patients with uncomplicated malaria, the most prescribed treatments were artemisinin-based combination therapy (ACT; n = 564 [94.3%]), primarily using artemether-lumefantrine (82.3%), in line with the World Health Organization (WHO) treatment guidelines. In addition, these patients received antipyretics (85.6%) and antibiotics (42.0%). However, in those with severe malaria, only 66 (48.2%) patients received parenteral treatment followed by oral ACT as per WHO guidelines, whereas 62 (45.3%) received parenteral treatment only. After receiving ambulatory care, 88.6% of patients with uncomplicated malaria were discharged and 83.2% of patients with severe malaria were discharged after hospitalization. One patient with uncomplicated malaria having multiple co-morbidities and three patients with severe malaria died.

**Conclusions:**

The findings of this study suggest that the prescribed treatment in most patients with uncomplicated malaria, but not of those with severe malaria, was in alignment with the WHO recommended guidelines.

**Supplementary Information:**

The online version contains supplementary material available at 10.1186/s12936-023-04650-y.

## Background

Malaria remains one of the most life-threatening diseases in sub-Saharan Africa. According to the latest World Malaria Report (WMR) 2022, globally there were an estimated 247 million cases in 2021 compared to 245 million cases in 2020, with the World Health Organization (WHO) African Region accounting for 95% of all malaria cases [1, 2]. Although malaria-related mortality rate reduced steadily between 2000 and 2019, there was a 10% increase in malaria deaths globally between 2019 and 2020 with an estimated 625,000 deaths followed by a slight decline in 2021 to 619,000 deaths. The WHO African Region contributes to 96% of deaths across all ages and 80% of deaths in children aged < 5 years [1, 2]. Between 2019 and 2021, there was an increase of 13 million malaria cases and 63,000 malaria deaths, primarily due to disruption in healthcare services caused by the ongoing coronavirus disease (COVID-19) pandemic [2]. Despite widespread deployment of a variety of recommended tools and strategies, malaria control has plateaued in recent years [3].

Early and correct diagnosis followed by prompt and effective treatment remains the key to controlling malaria. The WHO 2015 malaria guidelines, which were in force during the conduct of the study, recommend that all suspected cases of malaria should be confirmed through either microscopy or rapid diagnostic test (RDT) and treatment should begin in all confirmed cases soon after diagnosis [4]. For patients with uncomplicated malaria caused by *Plasmodium falciparum*, the WHO 2015 guidelines recommend a 3-day course of artemisinin-based combination therapy (ACT) except in pregnant women in their first trimester. The recommended treatment for pregnant women during the first trimester with uncomplicated malaria was quinine plus clindamycin for 7 days [4]. The WHO 2023 guidelines now recommend artemether-lumefantrine (AL) as an option for treatment of uncomplicated *Plasmodium falciparum* malaria during the first trimester of pregnancy [5]. In severe malaria, the recommendation is to use parenteral artesunate therapy for at least 24 h followed by a full 3-day course of oral treatment with an effective artemisinin-based combination across all demographics (including infants, pregnant women in all trimesters and lactating women). Since the risk of death from severe malaria is greatest in the first 24 h, either intramuscular artemether or parenteral quinine can be used when parenteral artesunate is not available [4, 5]. In hard-to-reach areas where parenteral artesunate is not available, pre-referral treatment of severe malaria in children aged < 6 years using a single dose of rectal artesunate, as suggested by the WHO, has shown significant reduction in mortality [6]. However, this approach was put on hold by the WHO in early 2022 based on the results of evidence from CARAMAL study until further guidance [6, 7]. In a recent review, it was concluded that many national malaria treatment guidelines for uncomplicated and severe malaria in non-endemic countries generally adhere to WHO recommendations. However, the choice between artemisinin-based combinations should be based on regional resistance patterns [8].

Although a substantial improvement has been made in prescribing treatments which align with recommendations from guidelines at healthcare facilities, there is a clear need for improved ways of educating patients and caregivers about the management of malaria, increased access to ACT, and compliance to guideline recommendations [9, 10]. Previous studies have reported low compliance (11–30%) to WHO treatment recommendations in managing patients with severe malaria. In a cross-sectional study in Swaziland between 2011 and 2015, patients with uncomplicated malaria were treated with quinine alone (5%), and AL alone was prescribed in 11% of patients with severe malaria [11]. Studies conducted at health facilities in Ethiopia, Ghana, and Uganda have reported that only 20–30% of patients have received treatment as per the recommendations from guidelines [12–14].

Data from different countries suggest that there is a gap between policies and local practices for diagnosis and treatment of malaria and these might vary across communities. To understand the management of malaria, it is critical to know the population characteristics, diagnostic approach, prescribing patterns and their alignment with the recommendations of WHO and local guidelines. The overall objective of this study was to evaluate the gap between guidelines and local clinical practice for the diagnosis and treatment of uncomplicated and severe malaria; and to describe the patient characteristics, diagnostic approach, and compliance of treatment to standard recommendations from guidelines.

## Methods

### Study design and data collection

**Fig. 1:**
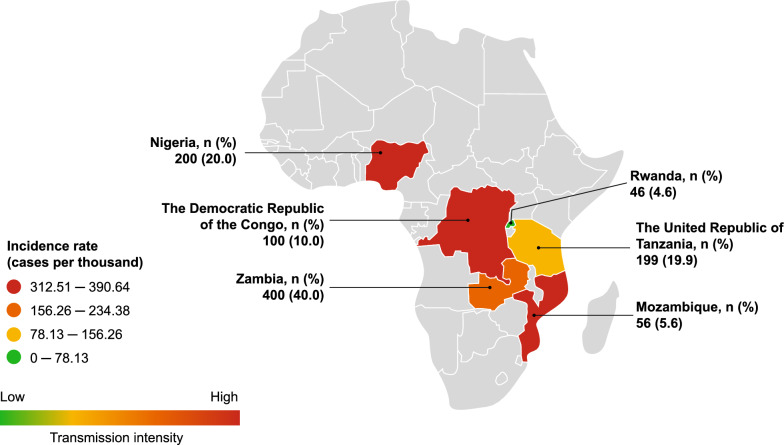
Incidence rate of malaria cases at study sites. Data from Malaria Atlas Project 2020 [15]

This was a prospective, multicentre, observational study that included infants, children, and adults of all ages with signs or symptoms suggestive of malaria who attended a healthcare facility across 11 sites in six countries from sub-Saharan Africa (The Democratic Republic of the Congo [DRC], Mozambique, Nigeria, Rwanda, The United Republic of Tanzania, and Zambia) between October 2020 and March 2021 (Fig. 1). Patients were considered to have confirmed malaria based on the overall clinical judgment by the investigator, irrespective of the outcome of diagnostic tests performed using RDT or microscopy. In this study, the patients were managed according to local standard practice and no specific interventions were required. Patients had an initial visit and a discharge visit that could occur on the same day. The baseline visit for the index malaria episode was when a patient presented at the study site seeking medical attention if malaria was confirmed. However, the study allowed for a follow-up of 30 days after the initial evaluation to collect data from any additional visit related to the patient’s index malaria episode, at the patient’s or the physician’s discretion. Individual patient-level records and prescriptions were collected for each patient through an electronic case report form at the study site. Data were also collected via a questionnaire provided to the patient or their legal guardian, which included socio-economic information and initial approach to disease at the community level.

### Study population

Patients of all age groups, including pregnant and breastfeeding women, who had fever (≥ 37.5 °C), or history of fever in the preceding 24 h, or other signs or symptoms suggestive of malaria were included. Patients participating in any other study (interventional clinical trial or observational study) at the time of enrolment were excluded.

### Study assessments

The primary assessment was proportion of patients receiving treatment prescriptions as per the WHO 2015 guidelines [4] among patients with a confirmed diagnosis of malaria, either uncomplicated or severe, in real-life settings in Africa. Secondary endpoints included demographic and socio-economic characteristics of patients and the proportion of patients who i) were tested with microscopy or malaria RDT for confirmation of malaria diagnosis and were classified as either uncomplicated or severe malaria, ii) self-medicated (drugstores or pharmacies), or received any traditional medicine (herbal or other), antibiotics or anti-malarial drugs before consulting the study site, and iii) were discharged and returned to the study site for follow-up within 30 days after initial discharge.

### Statistical analyses

All the analyses were performed descriptively. The number of observations (N), mean, median, standard deviation (SD), and minimum and maximum range were used for continuous variables. For categorical variables, N and percent were provided and the 95% confidence intervals (CIs) were provided as necessary. All analyses were performed by DATAMAP GmbH, Freiburg, Germany, using SAS Version 9.4. The estimated study sample size of approximately 1000 patients was determined based on the precision of the proportion estimator for each treatment defined by the half-width of the two-sided 95% CI. Patient enrolment was not stratified by subgroup, but analyses were also conducted for subgroups of age and pregnancy. The full analysis set (FAS) included all patients who signed the informed consent and provided at least the demographic data of age and gender. Confirmed malaria patients, either uncomplicated or severe, included patients in the FAS who were confirmed to have malaria based on overall clinical judgment by the investigator, irrespective of the outcome of diagnostic tests performed using RDT or microscopy.

## Results

### Patient disposition and baseline characteristics

A total of 1001 patients, comprising the FAS, were enrolled across 11 sites in six countries from sub-Saharan Africa: DRC, Mozambique, Nigeria, Rwanda, The United Republic of Tanzania, and Zambia. Of these, 981 (98%) patients completed the study. Reasons for discontinuation were death (n = 8), loss to follow-up (n = 5), physician decision (n = 6), and guardian decision (n = 1). The median age of patients was 11 years (range: 2 weeks–91 years) with 31.8% of patients aged < 5 years and 40.9% aged ≥ 18 years. Sixteen of the 37 pregnant women enrolled were in the third trimester. The demographic and baseline characteristics of patients are presented in Table 1.

**Table 1:** Demographic and baseline characteristics of patients

Characteristic	FAS (N = 1001)	Confirmed malaria (n = 735)	Confirmed malaria (n = 735)	
			Uncomplicated malaria (n = 598)	Severe malaria (n = 137)
Age (years)	17.6 (18.05)	18.3 (17.58)	19.8 (17.51)	11.6 (16.35)
Age group, n (%)				
1–28 days	3 (0.3)	2 (0.3)	1 (0.2)	1 (0.7)
29 days to 23 months	151 (15.1)	79 (10.7)	56 (9.4)	23 (16.8)
2–11 years	360 (36.0)	274 (37.3)	199 (33.3)	75 (54.7)
12–17 years	78 (7.8)	70 (9.5)	59 (9.9)	11 (8.0)
≥ 18 years	409 (40.9)	310 (42.2)	283 (47.3)	27 (19.7)
Second age group, n (%)				
< 5 years	318 (31.8)	195 (26.5)	129 (21.6)	66 (48.2)
5–11 years	196 (19.6)	160 (21.8)	127 (21.2)	33 (24.1)
≥ 12 years	487 (48.7)	380 (51.7)	342 (57.2)	38 (27.7)
Sex, n (%)				
Men	480 (48.0)	359 (48.8)	281 (47.0)	78 (56.9)
Women	521 (52.0)	376 (51.2)	317 (53.0)	59 (43.1)
Pregnant^a^	37 (7.1)	25 (6.6)	20 (6.3)	5 (8.5)
First trimester^b^	9 (24.3)	6 (24.0)	6 (30.0)	0
Second trimester^b^	12 (32.4)	11 (44.0)	8 (40.0)	3 (60.0)
Third trimester^b^	16 (43.2)	8 (32.0)	6 (30.0)	2 (40.0)
Race, n (%)				
Black or African American	1001 (100)	735 (100)	598 (100)	137 (100)
Ethnicity, n (%)				
Not Hispanic or Latino	1000 (99.9)	734 (99.9)	597 (99.8)	137 (100)
Not reported/unknown	1 (0.1)	1 (0.1)	1 (0.2)	0
Weight (kg)	38.0 (24.04)	38.0 (24.04)	41.5 (24.03)	23.7 (18.13)
BMI (kg/m^2^)	20.5 (6.20)	20.5 (6.20)	21.2 (6.30)	16.7 (3.80)

Data are presented as mean (SD) unless specified otherwise

*BMI* body mass index, *FAS* full analysis set

^a^Percentage based on number of women

^b^Percentage based on number of pregnant women

### Socio-economic characteristics

More patients who visited the study sites came from rural areas (44.9%) than from urban (30.6%) or suburban (24.5%) areas. For uncomplicated malaria, a greater proportion of patients were ≥ 12 years old (57.2%) and a smaller proportion of patients were < 5 years old (21.6%). Conversely, for severe malaria, a greater proportion of patients were < 5 years old (48.2%) and a smaller proportion of patients were aged ≥ 12 years old (27.7%). In patients presenting with severe malaria, a greater proportion were living in rural areas (57.7%) compared to suburban areas (6.6%) and a smaller number of patients completed secondary and higher education (14.6%–21.9%) compared to patients who completed primary education (48.9%). Socio-economic characteristics including employment and income status are presented in Table 2.

**Table 2:** Socio-economic characteristics of patients

Characteristics	FAS (N = 1001)	Confirmed malaria (n = 735)	Confirmed malaria (n = 735)	
			Uncomplicated malaria (n = 598)	Severe malaria (n = 137)
Questionnaire completed	1000	735	598	137
Status of residence, n (%)				
Urban	306 (30.6)	221 (30.1)	172 (28.8)	49 (35.8)
Suburb	245 (24.5)	193 (26.3)	184 (30.8)	9 (6.6)
Rural	449 (44.9)	321 (43.7)	242 (40.5)	79 (57.7)
Education level, n (%)				
None	80 (8.0)	40 (5.4)	20 (3.3)	20 (14.6)
Primary	359 (35.9)	258 (35.1)	191 (31.9)	67 (48.9)
Secondary	385 (38.5)	302 (41.1)	272 (45.5)	30 (21.9)
Higher	175 (17.5)	135 (18.4)	115 (19.2)	20 (14.6)
Missing	1 (0.1)	0	0	0
Employment status, n (%)				
Employed full time	139 (13.9)	105 (14.3)	90 (15.1)	15 (10.9)
Employed part time	73 (7.3)	60 (8.2)	47 (7.9)	13 (9.5)
Unemployed looking for work	85 (8.5)	71 (9.7)	70 (11.7)	1 (0.7)
Unemployed not looking for work	120 (12.0)	93 (12.7)	91 (15.2)	2 (1.5)
Self-employed	460 (46.0)	337 (45.9)	234 (39.1)	103 (75.2)
Retired	12 (1.2)	9 (1.2)	9 (1.5)	0
Homemaker	83 (8.3)	51 (6.9)	48 (8.0)	3 (2.2)
Unable to work	26 (2.6)	9 (1.2)	9 (1.5)	0
Missing	2 (0.2)	0	0	0
Income category, n (%)				
≤ poverty line	707 (70.7)	523 (71.2)	418 (69.9)	105 (76.6)
> 1 to < 2 poverty line	178 (17.8)	118 (16.1)	99 (16.6)	19 (13.9)
≥ 2 poverty line	114 (11.4)	94 (12.8)	81 (13.5)	13 (9.5)
Missing	1 (0.1)	0	0	0

*FAS* full analysis set

Percentages are based on the number of patients who completed the questionnaire

### Diagnosis and other assessments

**Fig. 2:**
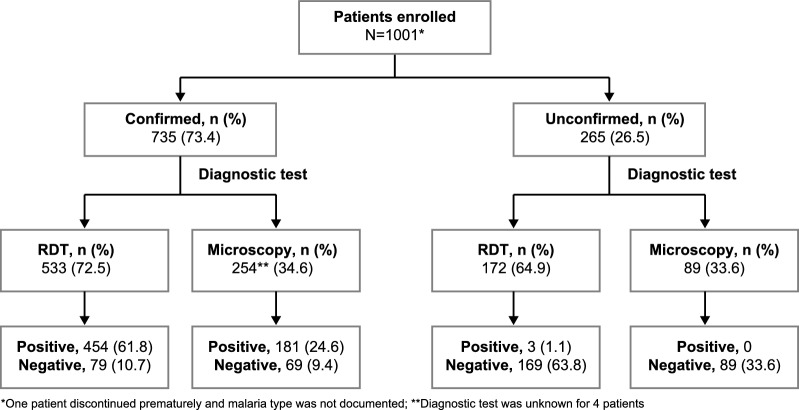
Participant flow along with their diagnostic test results. RDT, rapid diagnostic test

Overall, 735 (73.4%) patients had confirmed malaria (uncomplicated malaria [n = 598] and severe malaria [n = 137]) at baseline based on local evaluation. Cases were tagged as unconfirmed (26.5%) based on clinical judgment, including results from diagnostic tests and other evaluations. In patients with confirmed malaria, 533 (72.5%) were tested using RDT; of these, 454 patients had a positive result. Microscopy was performed in 254 (34.6%) patients; of these, 181 patients had a positive result (Fig. 2). Only a small percentage of patients (12 [1.6%]) had a diagnosis of clinically confirmed malaria without RDT or microscopy. Additionally, a small number of patients were diagnosed with malaria despite negative diagnostic tests (RDT [n = 79], microscopy [n = 69]).

**Fig. 3:**
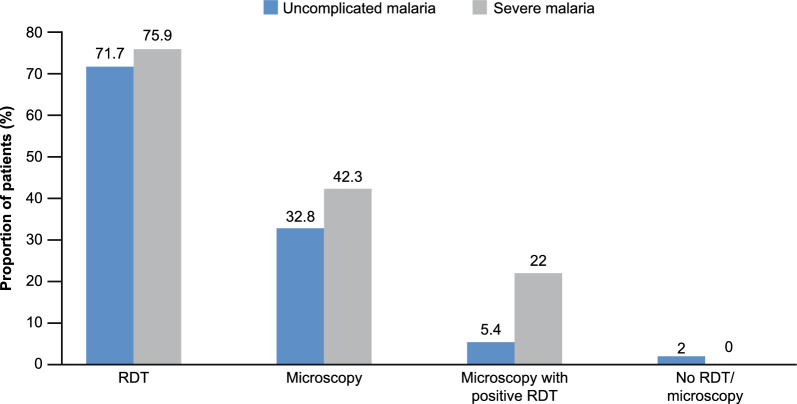
Proportion of patients diagnosed with different malaria diagnostic techniques. RDT, rapid diagnostic test

The proportions of patients administered with different diagnostic tests to confirm malaria, either uncomplicated or severe, are presented in Fig. 3. Overall, 79.7% of patients with confirmed malaria had vital signs performed at baseline. A greater proportion of patients with severe malaria underwent evaluations such as complete blood count (25.5%), blood glucose (21.9%), creatinine (12.4%), or blood urea nitrogen test (8.8%).

### Initial approach to malaria at community level

At community level, 51.8% of patients with uncomplicated malaria and 83.9% with severe malaria had sought advice or had received treatment for malaria, mostly from a drugstore or another health facility. This proportion was higher in patients with confirmed malaria (n = 418 [56.9%]) and unconfirmed malaria (n = 144 [54.3%]), who had taken one or more drugs, including anti-malarials and antibiotics, before coming to the study site (Additional file 1: Table S1). In terms of early access to care, 44.1% of patients enrolled into the study came to the study site for initial visit ≥ 48 h after symptom onset and 61.6% of patients travelled < 1 h from their residence to the study site (Additional file 1: Table S2).

### Treatment prescriptions

#### Uncomplicated malaria

**Fig. 4:**
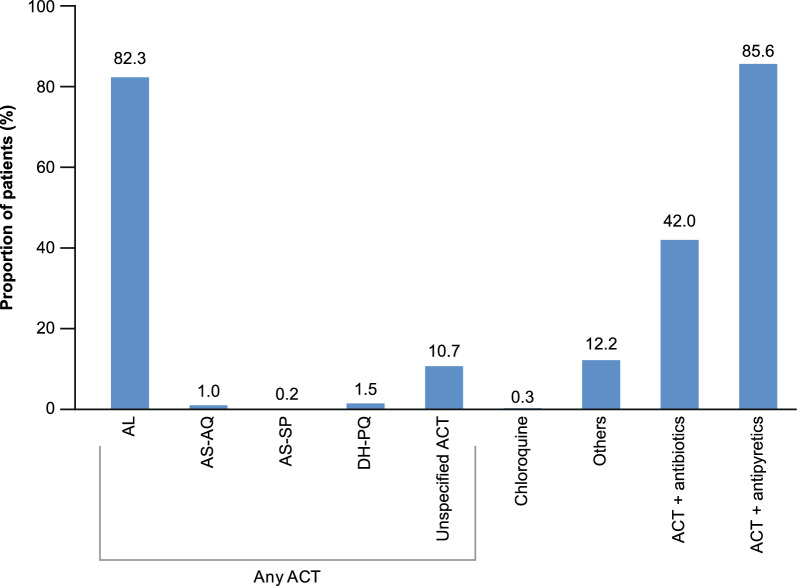
Treatment prescriptions for patients with uncomplicated malaria. *ACT* artemisinin-based combination therapy, *AL* artemether-lumefantrine, *AS-AQ* artesunate-amodiaquine, *AS-SP* artesunate-sulfadoxine pyrimethamine, *DH-PQ* dihydroartemisinin-piperaquine

In line with the WHO 2015 treatment guidelines, for patients diagnosed with uncomplicated malaria, the most prescribed treatments were ACT in 564 (94.3%) patients, primarily AL (82.3%), followed by an unspecified ACT (10.7%). In addition to ACT, patients also received antipyretics (85.6%) and antibiotics (42.0%) (Fig. 4). In children aged < 5 years, 20.2% of patients received ACT prior to the study site visit, and 78.3% received the ACT at the study site. AL remained the most common prescribed artemisinin-based combination in 65.1% of patients, followed by an unspecified ACT in 11.6% of patients. Of the 20 pregnant women, one received an unspecified ACT prior to the study site visit and 15 (75.0%) received ACT at the study site (AL [n = 14], artesunate and sulfadoxine-pyrimethamine [n = 1]), irrespective of their trimester. In infants < 5 kg (n = 5), three patients received antipyretics (66.6%) prior to the site visit and then received an ACT (100%), antipyretics (66.6%) and antibiotics (33.3%) at the study site. Two newborns received an unspecified ACT and antipyretic. Of note, in patients with negative diagnostic test, a higher proportion of patients received ACT (n = 94/102 [92.2%]) and 19.6% of patients received another treatment or combination of treatments.

#### Severe malaria

**Fig. 5:**
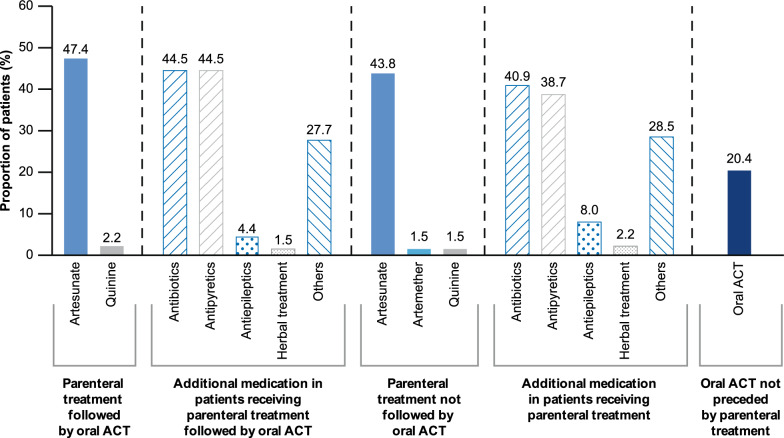
Treatment prescriptions for patients with severe malaria. *ACT* artemisinin-based combination therapy

Of a total of 137 patients with severe malaria, only 66 (48.2%) patients received parenteral treatment followed by an oral artemisinin-based combination as per the WHO 2015 guidelines, whereas 62 (45.3%) received parenteral treatment only (Fig. 5). Artesunate remained the most administered parenteral treatment in patients with severe malaria. However, there is a considerable difference in treatment prescriptions observed between patients with a positive (n = 124/137) or negative (n = 13/137) diagnostic test. Half of the patients (51.6%, 64/124) with a positive test received parenteral treatment followed by oral ACT compared to 15% (2/13) of patients with a negative test. In children aged < 5 years, 7.6% of patients received parenteral treatment not followed by oral ACT prior to the study site visit. At the study sites, 29 (43.9%) patients received parenteral treatment followed by oral ACT and 33 (50.0%) received parenteral treatment not followed by oral ACT (Additional file 1: Table S3). Across all study sites, none of the patients received rectal artesunate. Also, no pregnant women received treatment prior to the study site visit. Three pregnant women received parenteral treatment followed by oral ACT, while two received parenteral treatment only (Additional file 1: Table S3). One newborn received parenteral artesunate followed by oral ACT, antibiotics, and antipyretics. The treatment pattern in severe malaria cases by different countries is presented in Additional file 1: Table S4.

### Initial outcome and discharge status

The majority of patients with uncomplicated malaria (88.6%) were discharged after ambulatory care at the site, whereas 83.2% of patients with severe malaria were discharged after hospitalization. Four patients died during the study (one with uncomplicated malaria having comorbidities such as hypertension, asthma, thoracic and mediastinal disorders and sinusitis and three with severe malaria). Only 13 patients (2.2%) with uncomplicated malaria and 1 patient (0.7%) with severe malaria returned to the site within 30 days after initial discharge for follow-up.

## Discussion

In this study, the enrolment of patients was balanced across all ages. Of the patients confirmed to have malaria, 42% were aged ≥ 18 years and 6.6% were pregnant. Children aged < 5 years constituted only 26.5% of patients, which is lower than expected, but had a higher proportion of severe malaria cases (48.2%) compared to uncomplicated malaria (21.6%). The study was done in areas with varying malaria transmission intensity and the proportion of patients for different age groups is consistent with reports from other countries including Nigeria and Mozambique (Fig. 1). In highly endemic countries, the focus remains on traditional at-risk populations such as children aged < 5 years and pregnant women. Changes in malaria transmission intensity led to an age shift in the population at risk and outside high transmission areas there is a need to extend the focus of malaria control strategies to children aged > 5 years and adults [16].

The most commonly prescribed treatment for patients with uncomplicated malaria in this study was ACT in ~ 94% of patients, primarily AL, which is in line with WHO recommendations. For severe malaria, however, there was poor compliance with the latest WHO recommendations as only 48% of these patients received parenteral treatment followed by an oral ACT and less than half (40.9–44.5%) were initiated on antibiotics. The appropriate diagnosis of severe malaria can be challenging due to existing similarities in symptoms for severe malaria, pneumonia, and septicaemia. WHO guidelines for treating severe malaria also recommend rapid administration of parenteral artesunate and broad-spectrum antibiotic for simultaneous treatment of severe malaria, pneumonia, and septicaemia even before the availability of laboratory results to avoid fatal delays [5, 17].

Poor compliance to WHO treatment recommendations in patients with severe malaria has been observed previously in several studies [13, 18, 19]. A study conducted in Ghana and Uganda showed 30% compliance to WHO guidelines in patients with severe malaria [13]. Another case study in Uganda showed that only 16.9% of patients received appropriate treatment for severe malaria. The poor compliance was mainly due to sub-optimal healthcare facilities in Uganda [18]. A cross-sectional study in urban and rural areas in DRC showed that a higher proportion of patients living in urban areas (76.5%) adhered to treatment compared with those living in rural areas (66.8%) [19]. The level of education also played an important role in treatment compliance. Patients who were university graduates were more adherent than those who did not receive any education (78.3% vs. 68.2%) [19]. A recent retrospective study in Gambela town of Ethiopia showed that only one case of uncomplicated malaria was treated as per guideline recommendations, while 99.1% of cases did not receive treatment as per guidelines. These patients were treated with parenteral artesunate or quinine [14]. The reasons behind lack of compliance were not evaluated in the current study. However, some of the drivers for low compliance in previously published literature include poor access to healthcare facilities, limited diagnostic capacity, basic care package for severe malaria management at health centers, and lack of continuous medical education on the update in guideline recommendations [13, 14, 18, 19].

Of note, in this study, 92% of patients with a negative test result received an ACT and 19.6% received other treatment or combination of treatments for uncomplicated malaria based on clinical judgment. All malaria RDTs conducted in this study were *Plasmodium falciparum* histidine-rich protein 2 (PfHRP2)-based in alignment with national guidelines in Nigeria, DRC, Rwanda, Zambia, and Mozambique. In Tanzania, histidine-rich protein 2 (HRP-II)/pan-lactate dehydrogenase (pLDH)-based RDTs were used. In some cases, available local data was taken into consideration. A recent evaluation conducted in Mozambique found PfHRP2-based RDTs to still be appropriate for the diagnosis of malaria in that country [20]. Studies conducted in multiple regions of DRC have consistently found a low local prevalence of the mutation [21, 22]. Across the other countries in this study, while there is data to suggest presence of this mutation [23–27], the sample sizes were not always representative, and further work is recommended. None of these countries has switched to pLDH-based RDTs.

Prescribing anti-malarials to patients with negative malaria test results is common practice. Other studies have also shown a similar practice. One study in Kenya in 2016 reported that 69% of patients with a negative malaria test result received anti-malarial treatment [28]. In a retrospective study conducted at two public health facilities in Nigeria in patients with uncomplicated malaria, almost half of the patients (49%) had microscopy done and only half of these tested positive. Presumptive treatment was administered in 51% of patients. There was a disconnect between diagnosis and treatment prescription of anti-malarials in 93% of patients [9]. Another study in Ugandan children revealed that 39.6% of patients with negative test results were prescribed anti-malarial drugs [29]. In a retrospective study in Gambela town of Ethiopia, 96% of patients with negative results and 100% of untested patients received anti-malarial drugs [14]. While not evaluated in our study, some of the reasons driving this practice could be the high mortality associated with malaria and its extremely common occurrence, limited diagnostic capacity, lack of trust in negative test results, patient/caregiver pressure, and concerns about potentially fatal consequences on missing positive malaria cases. This highlights the need to utilize the diagnostic tests more efficiently and prescribe the treatment judiciously based on the results from diagnostic tests [30].

Of interest, patient recruitment in this study was done during the COVID-19 pandemic. But the pandemic did not really affect malaria care delivery at the study sites in DRC, Mozambique, Nigeria, Rwanda, and Zambia. However, the pandemic was speculated to have compromised the access, and delivery of health care services at different healthcare levels in Tanzania which may have led to a lower number of reported malaria cases and death. Also, there was a drop in health seeking behavior as patients were afraid of being infected if they visited the healthcare facilities [31]. During the post-pandemic period, the health-seeking behaviour is expected to improve. The study has several limitations. The qualitative data of patients were not collected; therefore, the factors driving behaviour of healthcare practitioner are not known. Sites were selected at random, and this could have led to potential bias in performing diagnosis before prescribing anti-malarials. Another limitation is that this analysis was limited to public healthcare facilities and there could be a difference in malaria treatment practices between the public and private healthcare settings.

## Conclusions

This study provides insights into health-seeking behaviour and treatment prescription practices in patients receiving treatment for malaria at healthcare facilities in sub-Saharan Africa. Overall, the study reveals that the prescribed treatment for most patients with uncomplicated malaria was in alignment with the WHO guideline recommendations. However, treatment of patients with severe malaria was found to be less compliant with the WHO-recommended approach of prescribing parenteral artesunate treatment followed by a full course of an effective oral artemisinin-based combination. Although high compliance to performing diagnostic tests was noted in patients with suspected malaria, there is a need to improve treatment prescriptions in patients with negative test results.

## Supplementary Information

**Additional file 1: Table S1**. Proportion of patients seeking prior advice or treatment before consulting healthcare facility. **Table S2**. Time elapsed in hours between first symptoms and start of journey to the study site. **Table S3**. Treatment prescriptions for patients with severe malaria in children aged < 5 years and pregnant women. **Table S4**. Treatment prescriptions for patients with severe malaria by different countries.

## Data Availability

The data collected during the study will be made available from the corresponding author on reasonable request.
