# Supplementary material for: Prescription patterns and compliance with World Health Organization recommendations for the management of uncomplicated and severe malaria: A prospective, real-world study in sub-Saharan Africa
Source: Malar J. 2023 Jul 25;22:215. doi: 10.1186/s12936-023-04650-y (PMC10367305; doi:10.1186/s12936-023-04650-y)
Supplement: Supplementary file 1 — Additional file 1: Table S1. Proportion of patients seeking prior advice or treatment before consulting healthcare facility. Table S2. Time elapsed in hours between first symptoms and start of journey to the study site. Table S3. Treatment prescriptions for patients with severe malaria in children aged < 5 years and pregnant women. Table S4. Treatment prescriptions for patients with severe malaria by different countries. [file 12936_2023_4650_MOESM1_ESM.docx]

**Table S1. Proportion of patients seeking prior advice or treatment before consulting healthcare facility**

| **Action before consulting study site** | **Confirmed malaria (n=735)** | **Confirmed malaria (n=735)** | | **Unconfirmed malaria (n=265)** |
| --- | --- | --- | --- | --- |
|  |  | **Uncomplicated malaria (n=598)** | **Severe malaria (n=137)** |  |
| **Any advice or treatment prior to coming to study site, n (%)** | 425 (57.8) | 310 (51.8) | 115 (83.9) | 146 (55.1) |
| Hospital / clinic | 86 (11.7) | 30 (5.0) | 56 (40.9) | 47 (17.7) |
| Private doctor | 20 (2.7) | 18 (3.0) | 2 (1.5) | 13 (4.9) |
| Community health worker | 4 (0.5) | 4 (0.7) | 0 | 3 (1.1) |
| Pharmacy | 62 (8.4) | 52 (8.7) | 10 (7.3) | 4 (1.5) |
| Over the counter drugstore | 92 (12.5) | 62 (10.4) | 30 (21.9) | 29 (10.9) |
| Traditional healer/practitioner | 2 (0.3) | 2 (0.3) | 0 | 2 (0.8) |
| Itinerant drug seller | 6 (0.8) | 5 (0.8) | 1 (0.7) | 5 (1.9) |
| None - self medication | 173 (23.5) | 149 (24.9) | 24 (17.5) | 41 (15.5) |
| Other | 11 (1.5) | 8 (1.3) | 3 (2.2) | 13 (4.9) |
| **Any drug taken during illness before coming to study site, n (%)** | 418 (56.9) | 306 (51.2) | 112 (81.8) | 144 (54.3) |
| **Antimalarials** | 159 (21.6) | 99 (16.6) | 60 (43.8) | 19 (7.2) |
| Artemisinin combination therapy | 125 (17.0) | 84 (14.0) | 41 (29.9) | 16 (6.0) |
| Sulfadoxine/pyrimethamine | 7 (1.0) | 6 (1.0) | 1 (0.7) | 1 (0.4) |
| Chloroquine | 2 (0.3) | 2 (0.3) | 0 | 0 |
| Amodiaquine | 4 (0.5) | 2 (0.3) | 2 (1.5) | 0 |
| Quinine | 11 (1.5) | 2 (0.3) | 9 (6.6) | 1 (0.4) |
| Oral | 4 (0.5) | 1 (0.2) | 3 (2.2) | 0 |
| IM/IV | 7 (1.0) | 1 (0.2) | 6 (4.4) | 1 (0.4) |
| Artesunate | 13 (1.8) | 2 (0.3) | 11 (8.0) | 3 (1.1) |
| Rectal | 0 | 0 | 0 | 0 |
| IM/IV | 13 (1.8) | 2 (0.3) | 11 (8.0) | 3 (1.1) |
| Other antimalarial | 6 (0.8) | 4 (0.7) | 2 (1.5) | 0 |
| **Antibiotics** | 73 (9.9) | 56 (9.4) | 17 (12.4) | 34 (12.8) |
| **Others** | 376 (51.2) | 277 (46.3) | 99 (72.3) | 138 (52.1) |
| Data are presented as n (%).  IM, intramuscular; IV, intravenous. | | | | |

**Table S2. Time elapsed in hours between first symptoms and start of journey to the study site**

| **Time elapsed** | **FAS (N=1001)** | **Confirmed malaria (n=735)** | **Confirmed malaria (n=735)** | |
| --- | --- | --- | --- | --- |
|  |  |  | **Uncomplicated malaria (n=598)** | **Severe malaria (n=137)** |
| First symptoms and start of journey to study site, n (%) | | | | |
| <8 h | 113 (11.3) | 74 (10.1) | 55 (9.2) | 19 (13.9) |
| 8 to <24 h | 233 (23.3) | 152 (20.7) | 122 (20.4) | 30 (21.9) |
| 24 to <48 h | 195 (19.5) | 142 (19.3) | 122 (20.4) | 20 (14.6) |
| ≥48 h | 441 (44.1) | 357 (48.6) | 290 (48.5) | 67 (48.9) |
| Unknown | 18 (1.8) | 10 (1.4) | 9 (1.5) | 1 (0.7) |
| Start of journey from place of residence and arrival at the study site, n (%) | | | | |
| <1 h | 617 (61.6) | 444 (60.4) | 387 (64.7) | 57 (41.6) |
| 1 to <3 h | 321 (32.1) | 248 (33.7) | 194 (32.4) | 54 (39.4) |
| 3 to <6 h | 43 (4.3) | 30 (4.1) | 13 (2.2) | 17 (12.4) |
| ≥6 h | 13 (1.3) | 10 (1.4) | 2 (0.3) | 8 (5.8) |
| Unknown | 6 (0.6) | 3 (0.4) | 2 (0.3) | 1 (0.7) |
| Data are presented as n (%).  FAS, full analysis set. | | | | |

**Table S3.** Treatment prescriptions for patients with severe malaria in children aged <5 years and pregnant women

| **Medication** | **Children <5 years (n=66)** | **Pregnant women (n=5)** |
| --- | --- | --- |
| **Parenteral treatment not followed by oral ACT, n (%)** | 33 (50.0) | 2 (40.0) |
| Artesunate | 33 (50.0) | 1 (20.0) |
| Artemether | 0 | 1 (20.0) |
| Quinine | 1 (1.5) | 0 |
| **Parenteral treatment followed by oral ACT, n (%)** | 29 (43.9) | 3 (60.0) |
| Artesunate followed by oral ACT | 29 (43.9) | 3 (60.0) |
| Artemether followed by oral ACT | 0 | 0 |
| Quinine followed by oral ACT | 1 (1.5) | 0 |
| **Oral ACT not preceded by parenteral treatment, n (%)** | 15 (22.7) | 0 |
| ACT, artemisinin-based combination therapies |  |  |

**Table S4.** Treatment prescriptions for patients with severe malaria by different countries

| **Medication** | **DRC (n=16)** | **Mozambique (n=2)** | **Nigeria (n=18)** | **Rwanda  (n=1)** | **United Republic of Tanzania (n=95)** | **Zambia (n=5)** |
| --- | --- | --- | --- | --- | --- | --- |
| **Parenteral treatment not followed by oral ACT, n (%)** | 10 (62.5) | 1 (50.0) | 14 (77.8) | 1 (100) | 36 (37.9) | 0 |
| Artesunate | 8 (50.0) | 1 (50.0) | 14 (77.8) | 1 (100) | 36 (37.9) | 0 |
| Artemether | 2 (12.5) | 0 | 0 | 0 | 0 | 0 |
| Quinine | 1 (6.3) | 0 | 0 | 0 | 1 (1.1) | 0 |
| **Parenteral treatment followed by oral ACT, n (%)** | 5 (31.3) | 0 | 3 (16.7) | 0 | 58 (61.1) | 0 |
| Artesunate followed by oral ACT | 5 (31.3) | 0 | 3 (16.7) | 0 | 57 (60.0) | 0 |
| Artemether followed by oral ACT | 0 | 0 | 0 | 0 | 0 (0.0) | 0 |
| Quinine followed by oral ACT | 0 | 0 | 0 | 0 | 3 (3.2) | 0 |
| **Oral ACT not preceded by parenteral treatment, n (%)** | 1 (6.3) | 1 (50.0) | 7 (38.9) | 0 | 14 (14.7) | 5 (100) |
| ACT, artemisinin-based combination therapies; DRC, The Democratic Republic of the Congo | | | | | | |
